# Open-label trial and randomized, double-blind, placebo-controlled, crossover trial of hydrogen-enriched water for mitochondrial and inflammatory myopathies

**DOI:** 10.1186/2045-9912-1-24

**Published:** 2011-10-03

**Authors:** Mikako Ito, Tohru Ibi, Ko Sahashi, Masashi Ichihara, Masafumi Ito, Kinji Ohno

**Affiliations:** 1 Division of Neurogenetics, Center for Neurological Diseases and Cancer, Nagoya University Graduate School of Medicine, 65 Tsurumai, Showa-ku, Nagoya 466-8550, Japan; 2 Faculty of Pathophysiology and Therapeutics, Aichi Medical University College of Nursing, 21 Karimata Yazako, Nagakute-cho, Aichi-gun, Aichi 480-1195, Japan; 3 Department of Neurology, Aichi Medical University School of Medicine, 21 Karimata Yazako, Nagakute-cho, Aichi-gun, Aichi 480-1195, Japan; 4 Department of Biomedical Sciences, College of Life and Health Sciences, Chubu University, 1200 Matsumoto, Kasugai, Aichi 487-8501, Japan; 5 Department of Longevity and Aging Research, Gifu International Institute of Biotechnology, 1-1 Nakafudogaoka, Kakamigahara, Gifu 504-0838, Japan

## Abstract

**Background:**

Molecular hydrogen has prominent effects on more than 30 animal models especially of oxidative stress-mediated diseases and inflammatory diseases. In addition, hydrogen effects on humans have been reported in diabetes mellitus type 2, hemodialysis, metabolic syndrome, radiotherapy for liver cancer, and brain stem infarction. Hydrogen effects are ascribed to specific radical-scavenging activities that eliminate hydroxyl radical and peroxynitrite, and also to signal-modulating activities, but the detailed molecular mechanisms still remain elusive. Hydrogen is a safe molecule that is largely produced by intestinal bacteria in rodents and humans, and no adverse effects have been documented.

**Methods:**

We performed open-label trial of drinking 1.0 liter per day of hydrogen-enriched water for 12 weeks in five patients with progressive muscular dystrophy (PMD), four patients with polymyositis/dermatomyositis (PM/DM), and five patients with mitochondrial myopathies (MM), and measured 18 serum parameters as well as urinary 8-isoprostane every 4 weeks. We next conducted randomized, double-blind, placebo-controlled, crossover trial of 0.5 liter per day of hydrogen-enriched water or placebo water for 8 weeks in 10 patients with DM and 12 patients with MM, and measured 18 serum parameters every 4 weeks.

**Results:**

In the open-label trial, no objective improvement or worsening of clinical symptoms was observed. We, however, observed significant effects in lactate-to-pyruvate ratios in PMD and MM, fasting blood glucose in PMD, serum matrix metalloproteinase-3 (MMP3) in PM/DM, and serum triglycerides in PM/DM. In the double-blind trial, no objective clinical effects were observed, but a significant improvement was detected in lactate in MM. Lactate-to-pyruvate ratios in MM and MMP3 in DM also exhibited favorable responses but without statistical significance. No adverse effect was observed in either trial except for hypoglycemic episodes in an insulin-treated MELAS patient, which subsided by reducing the insulin dose.

**Conclusions:**

Hydrogen-enriched water improves mitochondrial dysfunction in MM and inflammatory processes in PM/DM. Less prominent effects with the double-blind trial compared to the open-label trial were likely due to a lower amount of administered hydrogen and a shorter observation period, which implies a threshold effect or a dose-response effect of hydrogen.

## Background

Ohsawa and colleagues first reported an effect of hydrogen gas on cerebral infarction in June 2007 [1]. Effects of hydrogen administered in the forms of inhaled gas, drinking water, instillation, and intraperitoneal injection have been reported for 31, 4, and 5 diseases in animal models, cells, and humans, respectively [2]. Hydrogen exhibits prominent effects especially on oxidative stress-mediated diseases and inflammatory diseases in rodents. Hydrogen scavenges hydroxyl radicals and less efficiently peroxynitrite [1]. The radical-scavenging activities, however, are unlikely to be an exclusive mechanism, because the amount of radical oxygen species generated in rodents and humans is much more than the amount of hydrogen molecules taken up by the body. Indeed, the amount of hydrogen taken up by drinking hydrogen-enriched water (HEW) is 100 or more times less than that by inhaling 2% hydrogen gas, but drinking HEW exhibits beneficial effects as good as or even better than inhaling 2% hydrogen gas in rodents [2-4], which suggests the lack of a simple dose-response effect. Our previous study on type 1 allergy also indicates that hydrogen suppresses type 1 allergy by acting as a gaseous signal modulator not as a radical scavenger [5].

Effects of hydrogen in humans have been examined in five studies. First, a randomized, double-blind, placebo-controlled crossover study of 900 ml/day of HEW for 8 weeks in 30 patients with diabetes mellitus type 2 demonstrated significant decreases of electronegative charge-modified LDL cholesterol, small dense LDL, and urinary 8-isoprostanes [6]. Second, an open-label trial of electrolyzed hydrogen-enriched hemodialysis solution in 9 patients for 4 months [7] and 21 patients for 6 months [8] showed significant decreases of systolic blood pressure before and after dialysis, as well as of plasma monocyte chemoattractant protein 1 and myeloperoxidase. Third, an open-label trial of 1.5-2.0 liters per day of HEW for 8 weeks in 20 subjects with metabolic syndrome exhibited a 39% increase of urinary superoxide dismutase (SOD), a 43% decrease of urinary thiobarbituric acid reactive substances (TBARS), an 8% increase of high density lipoprotein (HDL)-cholesterol, and a 13% decrease of total cholesterol/HDL-cholesterol ratio [9]. Fourth, a randomized placebo-controlled study of 1.5-2.2 liters/day of HEW for 6 weeks in 49 patients receiving radiotherapy for malignant liver tumors showed marked improvements of QOL scores [10]. As the study was not blinded, subjective QOL scores tended to be overestimated by a placebo effect, but objective markers for oxidative stress were also significantly decreased. Fifth, drip infusion of hydrogen-enriched saline in combination with Edaravone, a clinically approved radical scavenger for cerebral infarction, for 7 days in 8 patients with brain stem infarction was compared to 24 such patients receiving Edaravone alone [11]. Although the study was not randomized and not blinded, MRI markers of patients infused with hydrogen showed significant improvements and accelerated normalization.

Being prompted by the prominent effects of hydrogen on inflammatory diseases and oxidative stress-mediated diseases especially in rodents, we performed an open-label trial of drinking 1.0 liter per day of HEW for 12 weeks in 14 patients with muscle diseases, and identified improvement in four parameters: (i) a decrease of the lactate-to-pyruvate ratio in mitochondrial myopathies (MM) and progressive muscular dystrophy (PMD); (ii) a decrease of serum matrix metalloproteinase-3 (MMP3) in polymyositis/dermatomyositis (PM/DM), (iii) a decrease of fasting glucose in PMD, and (iv) a decrease of serum triglycerides in PM/DM. We then conducted a randomized, double-blind, placebo-controlled, crossover trial of 0.5 liter per day of HEW for 8 weeks in 12 MM and 10 DM cases. We observed that HEW significantly improved serum lactate in MM. In both studies, some patients reported subjective improvement of fatigability, diarrhea, and myalgia, but others reported floating sensation and worsening of diarrhea. We observed no objective improvement or worsening of clinical symptoms during each study. Our studies imply that HEW improves clinical parameters in MM and PM/DM, but 0.5 liter/day for 8 weeks is likely to be insufficient to demonstrate statistically significant effects.

## Patients and methods

### Patients

For the open-label trial, we recruited 5 patients with PMD, 4 patients with PM/DM, and 5 patients with MM. The PMD patients comprised 1 male with Miyoshi myopathy and 4 females with limb girdle muscular dystrophy type 2B with an average age and SD of 50.4 ± 15.9 years (range 25 - 66). The PM/DM patients comprised 2 males and 2 females with an average age of 53.8 ± 24.8 years (range 29 - 83). All the PM/DM cases were taking 5 - 10 mg of prednisolone per day and were well controlled. The MM patients comprised 4 cases with MELAS (2 males and 2 females with an average age of 45.8 ± 12.3 years, range 37 - 64) and a 54-year-old female with chronic progressive external ophthalmoplegia (CPEO).

For the randomized, double-blind, placebo-controlled, crossover trial, we recruited 12 patients with MM and 10 patients with DM. The MM patients comprised 5 cases with MELAS (2 males and 3 females with an average age of 44.6 ± 17.6 years, range 20 - 65), as well as 7 cases with CPEO (3 males and 4 females with an average age of 49.1 ± 11.1 years, range 29 - 61). The DM patients comprised 3 males and 7 females with an average age of 49.6 ± 13.7 years (range 32 - 66). All the DM patients were well controlled with 5 - 10 mg prednisolone per day. Three MM and three DM patients participated in both trials. Both trials were approved by the Ethical Review Board of the Aichi Medical University. Informed consent was obtained from each patient.

### Protocols

We purchased 500 ml HEW or placebo water in aluminum pouch from Blue Mercury Inc. (Tokyo, Japan). We measured hydrogen concentrations using an H2-N hydrogen needle sensor attached to a PA2000 2-Channel Picoammeter (Unisense Science, Aarhus, Denmark). The hydrogen concentrations were ~0.5 ppm (~31% saturation). We also confirmed that hydrogen in placebo water was undetectable with our system. For each trial, we instructed patients to evacuate the air from the pouch and to close a plastic cap tightly every time after they drink water to keep the hydrogen concentration as high as possible.

For the open-label trial, patients took 1.0 liter per day of HEW in five to ten divided doses for 12 weeks. We measured 18 serum and one urinary parameters and recorded clinical symptoms at 0, 4, 8, 12, 16 weeks.

For the double-blind trial, patients took 0.5 liter per day of HEW or placebo water in two to five divided doses for 8 weeks. Between the 8-week trials with HEW and placebo, we placed a 4-week washout period. We measured 18 serum parameters and recorded clinical symptoms at 0, 4, 8, 12, 16, 20, 24 weeks. In the double-blind trial, we did not measure urinary 8-isoprostane levels.

The data were statistically analyzed using one-way repeated measures ANOVA for the open-label trial and two-way repeated measures ANOVA for the double-blind trial, both followed by the Bonferroni's multiple comparison test using Prism version 4.0c (Graphpad Software, San Diego, CA).

## Results

### Open-label trial

**Figure 1:**
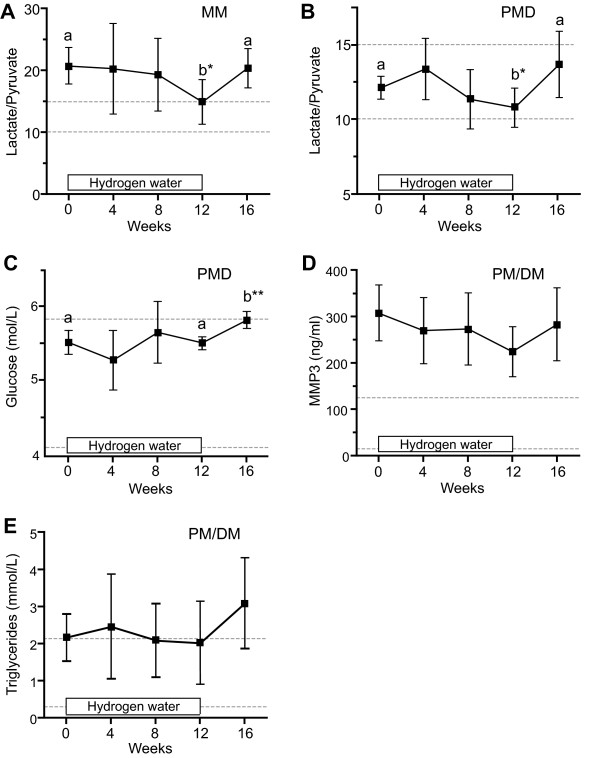
**Temporal profiles of four parameters that demonstrate statistical significance by one-way repeated measures ANOVA in the open-label trial**. Ratios of serum lactate/pyruvate (L/P) in 5 mitochondrial myopathies (MM) patients **(A) **and 4 progressive muscular dystrophy (PMD) patients **(B)**. Note abnormally high L/P ratios in MM patients. **(C) **Fasting glucose in 4 PMD patients. **(D) **Serum MMP3 in 5 Polymyositis (PM)/Dermatomyositis (DM) patients. **(E) **Serum triglycerides in 4 PMD patients. Twelve weeks on HEW are indicated by a box in each panel. Means and SD are plotted. Statistically different values by the Bonferroni's multiple comparison test are indicated by 'a' and 'b' with *p < 0.05 and **p < 0.01. Bonferroni's test reveal no statistical difference between any two values in **(D) **and **(E)**. Broken lines show a normal range of each parameter.

Fourteen patients with PMD, PM/DM, and MM participated in the study and no patient dropped out of the study. Patients took 1.0 liter of HEW for 12 weeks and we measured 18 serum and one urinary parameters every 4 weeks (Table 1). We observed no objective improvement or worsening of clinical symptoms during the study. All the patients reported increased micturition frequency. Two MELAS patients reported improvement of fatigability, and another MELAS patient complained mild occasional floating sensation. We estimated statistical significance using one-way repeated measures ANOVA analysis and detected five parameters (Figure 1). Serum lactate-to-pyruvate (L/P) ratios of MM patients were high before the study, and were decreased during the study (Figure 1A). Serum L/P ratios and fasting glucose levels of PMD patients were elevated after the study, but the values were still within normal ranges (Figures 1B and 1C). Serum MMP3 levels of DM patients were decreased down to 72.9% of those before HEW, which were again increased after the study (Figure 1D). Serum triglyceride levels of DM patients were elevated after the study (Figure 1E).

**Table 1:** Open-label trial of HEW in 14 myopathic patients

	Progressive muscular dystrophy (PMD)			Polymyositis (PM)/Dermatomyositis (DM)			Mitochondrial myopathies (MM)		
	
	Before	12 weeks	After	Before	12 weeks	After	Before	12 weeks	After
CK (U/L)	3067 ± 1492	3419 ± 1610	3107 ± 2382	124 ± 31	180 ± 97	140 ± 86	187 ± 75	124 ± 47	156 ± 40
HbA1c (%)	5.25 ± 0.44	5.14 ± 0.31	5.16 ± 0.42	6.68 ± 1.61	6.70 ± 1.53	6.90 ± 2.03	7.40 ± 1.70	7.32 ± 1.48	7.38 ± 1.74
Fasting glucose (mmol/L)	5.52 ± 0.16**	5.51 ± 0.08**	5.82 ± 0.11**	7.66 ± 0.11	7.29 ± 1.57	7.69 ± 1.85	8.94 ± 3.24	9.31 ± 4.18	8.96 ± 3.19
Lactate (mmol/L)	0.95 ± 0.34	1.15 ± 0.40	1.35 ± 0.40	1.42 ± 0.18	1.66 ± 0.32	1.30 ± 0.27	1.84 ± 0.50	1.87 ± 0.78	1.73 ± 0.65
L/P ratio	12.1 ± 0.7*	10.7 ± 1.3*	13.6 ± 2.2*	13.1 ± 0.9	15.0 ± 3.2	12.7 ± 1.0	20.7 ± 2.9*	14.9 ± 3.5*	20.3 ± 3.1*
Creatinine (μmol/L)	34.8 ± 3.1	34.5 ± 6.9	34.7 ± 8.8	58.6 ± 13.7	56.3 ± 10.7	56.6 ± 14.1	48.8 ± 9.0	47.7 ± 9.7	48.6 ± 8.8
BUN (mmol/L)	4.74 ± 1.16	4.20 ± 0.60	4.21 ± 1.05	4.33 ± 0.71	4.11 ± 0.48	4.68 ± 0.74	5.28 ± 1.69	5.89 ± 1.09	5.00 ± 1.58
Uric acid (μmol/dL)	295 ± 46	315 ± 61	300 ± 30	319 ± 44	331 ± 71	329 ± 40	208 ± 50	220 ± 60	217 ± 45
Urinary 8-isoprostane (ng/mg Cr)	303 ± 155	392 ± 173	n.d.	222 ± 88	237 ± 86	n.d.	274 ± 117	261 ± 59	n.d.
T-chol (mmol/L)	5.42 ± 0.99	5.74 ± 1.02	5.70 ± 0.81	5.28 ± 0.31	5.55 ± 0.93	5.97 ± 1.35	4.52 ± 0.75	4.53 ± 0.34	4.42 ± 0.77
LDL-chol (mmol/L)	3.30 ± 1.05	2.86 ± 1.46	2.99 ± 1.15	2.66 ± 0.28	3.21 ± 0.99	3.13 ± 1.02	2.44 ± 0.43	2.27 ± 0.44	2.22 ± 0.45
HDL-chol (mmol/L)	1.62 ± 0.21	1.49 ± 0.19	1.62 ± 0.18	2.01 ± 0.73	1.99 ± 0.76	2.02 ± 0.67	1.14 ± 0.79	1.03 ± 0.72	1.04 ± 0.69
Triglycerides (mmol/L)	1.31 ± 0.46	3.62 ± 4.83	3.07 ± 3.67	2.17 ± 0.63*	2.01 ± 1.21^* ^	3.09 ± 1.22*	0.78 ± 0.34	0.89 ± 0.45	0.73 ± 0.37
WBC (10^9^/L)	5.30 ± 1.32	5.40 ± 1.12	4.80 ± 0.38	10.78 ± 2.08	8.35 ± 3.41	10.10 ± 0.17	5.12 ± 1.27	7.27 ± 2.29	5.93 ± 1.35
RBC (10^12^/L)	4.17 ± 0.64	3.79 ± 0.42	3.87 ± 0.80	4.01 ± 0.62	4.34 ± 0.36	4.49 ± 0.45	4.33 ± 0.43	4.31 ± 0.78	4.35 ± 0.59
Platelets (10^9^/L)	262 ± 42	260 ± 33	270 ± 20	337 ± 123	265 ± 82	270 ± 65	215 ± 25	207 ± 28	217 ± 25
Hematocrit	0.375 ± 0.040	0.374 ± 0.038	0.407 ± 0.055	0.337 ± 0.069	0.376 ± 0.041	0.395 ± 0.045	0.381 ± 0.036	0.370 ± 0.047	0.378 ± 0.038
MMP3 (ng/ml)	n.d.	n.d.	n.d.	307.8 ± 59.1*	224.3 ± 53.4*	283.3 ± 77.6*	n.d.	n.d.	n.d.
IgG (mg/dl)	n.d.	n.d.	n.d.	1343 ± 470	1396 ± 550	1429 ± 581	n.d.	n.d.	n.d.

Values represent mean ± SD. n.d., not determined. *p < 0.05 and **p < 0.005 by one-way repeated measures ANOVA.

### Randomized, double-blind, placebo-controlled, crossover trial

**Figure 2:**
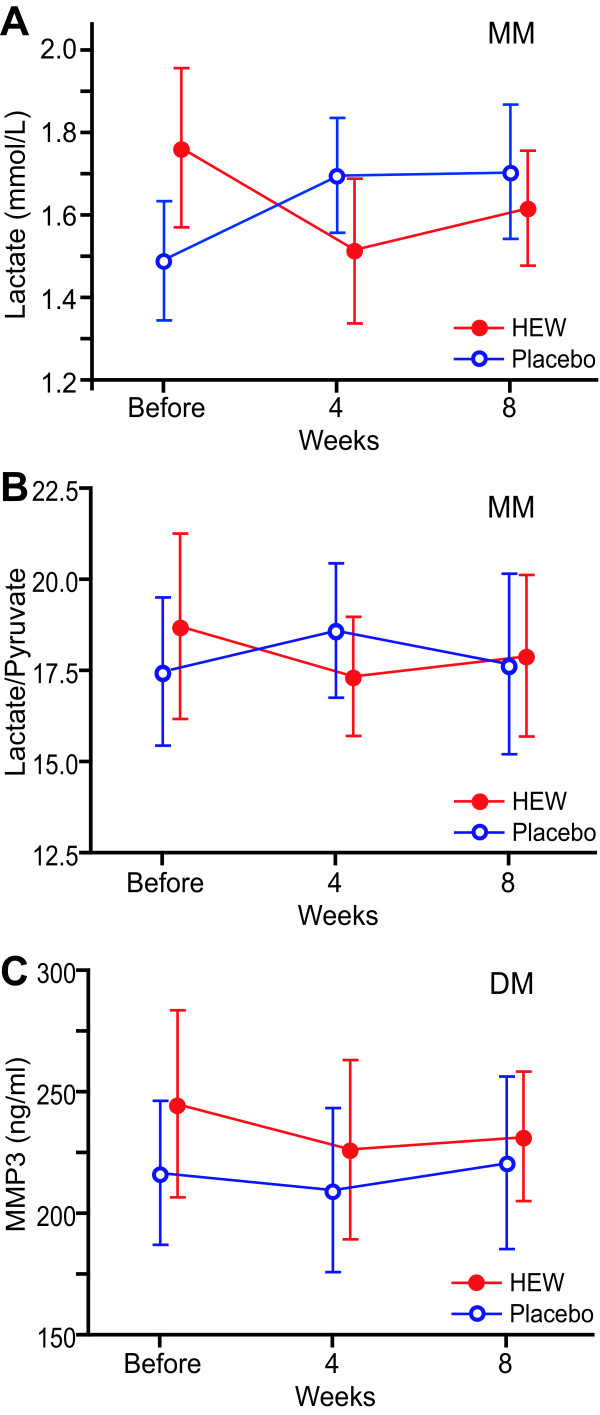
**Temporal profiles of three parameters in the double-blind trial**. Serum lactate **(A) **and L/P ratios **(B) **in 12 mitochondrial myopathies (MM) patients. **(C) **Serum MMP3 in 10 dermatomyositis (DM) patients. Patients took HEW or placebo for 8 weeks. Means and SD are plotted. **(A) **Serum lactate levels in MM are statistically different by two-way repeated measures ANOVA (p < 0.05, Table 1), but the Bonferroni's multiple comparison test reveals no statistical difference between any two values. No statistical difference was observed in L/P ratios **(B) **and serum MMP3 **(C)**. The normal range of lactate is 0.5-2.2 mmol/l. Normal ranges of the other parameters are indicated in Figure 1.

Twelve MM and ten DM patients participated in the study and no patient dropped out of the study. Patients took 0.5 liter of HEW or placebo water for 8 weeks and we measured 18 serum parameters every 4 weeks (Table 2). An MM patient reported increased micturition frequency on HEW. A DM patient reported subjective improvement of fatigability and diarrhea on HEW, but an MM patient rather complained increased diarrhea at first on HEW. Another DM patient reported an improvement of myalgia on HEW. A MELAS patient had hypoglycemic episodes only on HEW, but the episodes subsided after the insulin dose was decreased. We observed no objective improvement or worsening of clinical symptoms during the study. Two-way repeated measures ANOVA analysis revealed that only serum lactate levels were significantly decreased in MM by HEW (Figure 2A). Temporal profiles of serum L/P ratios in MM (Figure 2B) and of serum MMP3 levels in DM (Figure 2C) also demonstrated favorable responses to HEW but without statistical significance.

**Table 2:** Randomized, double-blind, placebo-controlled, crossover trial of HEW in 10 DM and 12 MM patients

	Dermatomyositis (DM)				Mitochondrial myopathies (MM)			
	
	Hydrogen water		Placebo water		Hydrogen water		Placebo water	
	
	0 week	8 weeks	0 week	8 weeks	0 week	8 weeks	0 week	8 weeks
CK (U/L)	88.7 ± 24.3	106.1 ± 88.2	93.5 ± 45.0	99.5 ± 86.7	165 ± 86.6	120 ± 55.5	142.0 ± 69.4	221 ± 235
HbA1c (%)	6.23 ± 1.28	6.34 ± 1.55	6.27 ± 1.44	6.16 ± 1.28	6.09 ± 0.94	6.12 ± 1.05	6.06 ± 1.22	6.06 ± 1.02
Fasting glucose (mmol/L)	8.27 ± 3.62	7.81 ± 2.91	6.70 ± 2.11	6.48 ± 1.90	6.05 ± 1.43	5.67 ± 1.99	6.02 ± 1.46	6.11 ± 1.69
Lactate (mmol/L)	1.93 ± 0.78	1.81 ± 0.87	1.80 ± 0.89	1.65 ± 0.77	1.76 ± 0.67*	1.61 ± 0.48*	1.49 ± 0.49*	1.70 ± 0.57*
L/P ratio	13.1 ± 6.0	11.5 ± 2.6	12.1 ± 2.88	15.2 ± 8.3	18.7 ± 8.8	17.9 ± 7.7	7.12 ± 13.4	17.7 ± 8.6
Creatinine (μmol/L)	59.1 ± 15.6	59.1 ± 13.6	58.3 ± 15.0	59.0 ± 19.7	53.6 ± 18.3	52.0 ± 17.4	54.8 ± 20.3	57.5 ± 22.3
BUN (mmol/L)	5.36 ± 1.48	4.82 ± 1.57	5.78 ± 1.71	4.86 ± 1.56	6.08 ± 2.09	5.39 ± 1.54	6.24 ± 1.46	6.34 ± 2.76
Uric Acid (μmol/dL)	303 ± 88	320 ± 68	313 ± 75	321 ± 83	413 ± 316	375 ± 229	447 ± 387	408 ± 284
T-chol (mmol/L)	5.23 ± 0.78	5.15 ± 0.85	5.21 ± 0.61	4.95 ± 0.92	4.61 ± 0.78	4.80 ± 0.57	4.69 ± 0.78	4.68 ± 0.71
LDL-chol (mmol/L)	3.06 ± 0.82	2.93 ± 0.75	2.97 ± 0.80	3.03 ± 0.84	2.69 ± 0.68	2.82 ± 0.58	2.73 ± 0.67	2.79 ± 0.60
HDL-chol (mmol/L)	1.59 ± 0.48	1.56 ± 0.36	1.53 ± 0.47	1.43 ± 0.48	1.47 ± 0.04	1.55 ± 0.34	1.52 ± 0.34	1.45 ± 0.35
Triglycerides (mmol/L)	1.54 ± 0.65	1.83 ± 0.76	1.86 ± 0.80	1.85 ± 0.48	1.16 ± 0.58	0.92 ± 0.35	0.97 ± 0.36	1.02 ± 0.49
WBC (10^9^/L)	11.7 ± 6.4	10.9 ± 3.1	9.66 ± 2.68	11.2 ± 4.7	5.5 ± 0.1	5.7 ± 1.8	5.81 ± 1.78	5.8 ± 1.9
RBC (10^12^/L)	4.28 ± 0.05	4.32 ± 0.35	4.37 ± 0.44	4.35 ± 0.44	4.15 ± 0.04	4.17 ± 0.61	4.26 ± 0.67	4.15 ± 0.56
Platelets (10^9^/L)	271 ± 10	302 ± 69	306 ± 54	312 ± 79	203 ± 40	214 ± 44	216 ± 48	225 ± 50
Hematocrit (%)	0.380 ± 0.004	0.384 ± 0.040	0.392 ± 0.052	0.391 ± 0.047	0.373 ± 0.004	0.378 ± 0.060	0.386 ± 0.063	0.376 ± 0.058
MMP3 (ng/ml)	245 ± 122	232 ± 84.1	217 ± 93.5	221 ± 112	n.d.	n.d.	n.d.	n.d.
IgG (mg/dl)	1211 ± 357	1244 ± 305	1202 ± 340	1282 ± 353	n.d.	n.d.	n.d.	n.d.

Values represent mean ± SD. n.d., not determined. *p < 0.05 by two-way repeated measures ANOVA.

## Discussion

We performed open-label and double-blind studies of HEW on myopathic patients. In the open-label study, we observed statistical significance of hydrogen effects in four parameters: L/P ratios in MM and PMD; fasting glucose in PMD; MMP3 in PM/DM; and triglycerides in PM/DM (Figure 1). In the double-blind study, serum lactate levels were significantly improved in MM. L/P ratios in MM and MMP3 in DM were also improved but without statistical significance (Figure 2). Small numbers of participants in both the open-label and double-blind studies might have failed to disclose statistically significant effects of HEW.

In MM, the mitochondrial electron transfer system (mETS) is compromised by mutations in mitochondrial DNA [12]. This results in a decreased influx of NADH into mETS and elevates NADH levels in the cytoplasm, which facilitates conversion of pyruvate to lactate by lactate dehydrogenase. Thus, lactate and L/P ratio are useful surrogate markers to estimate functions of mETS, and are usually abnormally elevated in MM [12]. Defective mETS also causes leakage of electrons from mitochondrial inner membranes and increases production of reactive oxygen species (ROS), which further damages mETS [13,14]. Reduction of the L/P ratios in the open-label and double-blind studies suggests that hydrogen alleviates mETS dysfunction in MM either by scavenging ROS or by yet unidentified signaling mechanisms.

MMP3 belongs to a family of calcium-dependent zinc proteinases induced by cytokines and secreted by inflammatory cells. MMPs enhance T-cell migration and adhesion, and also degrade the extracellular matrix proteins [15]. MMP3 is increased in a fraction of DM patients [16]. MMP3 may facilitate lymphocyte adhesion and enhance T-cell-mediated cytotoxicity by degrading extracellular matrix proteins in DM. Hydrogen improved serum MMP3 levels in the open-label and double-blind studies, which is expected to ameliorate pathogenic inflammatory processes that culminates in muscle fiber destruction.

We observed less prominent effects with the double-blind study compared to the open-label study. The lack of statistically significance in the double-blind study is possibly due to a lower amount of HEW (1.0 vs. 0.5 liter per day) and to a shorter observation period (12 vs. 8 weeks). In the open-label study, drinking 1.0 liter of HEW was not readily accommodated by most myopathic patients. Hydrogen does not show simple dose-response relationship in rodents [2-4], and *ad libitum *administration of even 5%-saturated HEW markedly attenuates development of Parkinson's disease in mice [17]. We thus reduced the amount of hydrogen to 0.5 liter in the double-blind trial, and also shortened the observation period to minimize the burden on the participants. This, however, might have masked effects of HEW. Indeed, when we compare studies of diabetes mellitus type 2 [6], the current open-label trial, and metabolic syndrome [9], the participants took 0.9, 1.0, and 1.5-2.0 liters of HEW, respectively. Ratios of total cholesterol/HDL-cholesterol are available at 8 weeks in all the studies, and are changed to 103.8%, 98.6%, and 95.8%, respectively, of those before hydrogen administration, which is in accordance with a dose-response effect of HEW. Additionally, among the two previous studies [6,9] and the current open-label and double-blind studies, the most prominent effects are observed with 1.5-2.0 liters of HEW. As drinking a large amount of HEW is not easily accommodated by most patients especially in winter, a threshold effect and/or a dose-response effect should be further elaborated for each pathological state.

## Conclusions

HEW is effective for mitochondrial dysfunction in MM and inflammatory processes in DM. Hydrogen may have a threshold effect or a dose-response effect and 1.0 liter or more per day of HEW is likely to be required to exert beneficial effects.

## Abbreviations

HEW: hydrogen-enriched water; PMD: progressive muscular dystrophy; PM: polymyositis; DM: dermatomyositis; MM: mitochondrial myopathies; CPEO: chronic progressive external ophthalmoplegia; MELAS: mitochondrial myopathy with lactic acidosis and stroke-like episodes; MMP3: matrix metalloproteinase-3.

## Competing interests

The authors declare that they have no competing interests.

## Authors' contributions

TI and KS examined patients and acquired data. MI^1 ^and TI organized data and performed statistical analysis. MI^1 ^and KO wrote the paper. MI^4^, MI^5^, and KO conceived the study. All authors read and approved the final manuscript.
